# Application of 3D-printed osteotomy guide plates in proximal femoral osteotomy for DDH in children: a retrospective study

**DOI:** 10.1186/s13018-023-03801-w

**Published:** 2023-04-24

**Authors:** Jian Sun, Yulei Mu, Yong Cui, Jing Qu, Feng Lian

**Affiliations:** Department of Orthopaedic Surgery, The Fourth Affiliated Hospital of Harbin Medical University, No. 37, Yiyuan Street, Nangang District, Harbin City, 150001 Heilongjiang Province China

**Keywords:** 3D-printed, Osteotomy guide plate, Developmental dysplasia of the hip, Femoral osteotomy

## Abstract

**Background:**

Patients with developmental dysplasia of the hip (DDH) have complex proximal femoral deformities, and orthopedic surgery lacks objectivity. Expectations for surgical outcomes are often not achieved, and postoperative problems are common. Using 3D-printed technology in orthopedics offers a novel approach to precise and individualized treatment in modern orthopedics. The aim of this study was to investigate the value of the application of 3D-printed osteotomy guide plates in femoral osteotomy. The clinical indices of femoral osteotomy in children with DDH using 3D-printed osteotomy guide plates were compared with those of traditional osteotomy.

**Methods:**

The clinical data of children with DDH who underwent open reduction and Salter pelvic osteotomy combined with femoral osteotomy from September 2010 to September 2020 were retrospectively collected and analyzed. Based on the inclusion and exclusion criteria, a total of 36 patients were included in the study: 16 in the guide plate group and 20 in the conventional group. Operation time (total), operation time (femoral side), X-ray fluoroscopy times (total), X-ray fluoroscopy times (femoral side) and intraoperative blood loss were analyzed and compared between the two groups. Comparison of treatment-related indicators such as postoperative neck-shaft angle, postoperative anteversion angle, hospitalization time, and hospitalization expenses is made between the two groups. The two groups of patients were evaluated at the last follow-up using the McKay clinical evaluation criteria.

**Results:**

Between the two groups, there were significant differences in operation time (total), operation time (femoral side), X-ray fluoroscopy times (total), X-ray fluoroscopy times (femoral side) and intraoperative blood loss (*P* < 0.05). The postoperative neck-shaft angle, postoperative anteversion angle, hospitalization time and hospitalization expenses did not differ significantly (*P* > 0.05). The MacKay clinical evaluation did not significantly differ at the most recent follow-up (*P* > 0.05).

**Conclusions:**

Children with DDH undergoing proximal femoral osteotomy using 3D-printed osteotomy guide plates benefit from a simpler surgical procedure, shorter operative time, less bleeding and less radiation exposure during surgery. This technique is of great clinical value.

## Introduction

Hip dysplasia, subluxation and complete dislocation are some of the hip deformities included in developmental dysplasia of the hip (DDH), a common developmental deformity of the hip in infants and children [1, 2]. Patients often have structural changes in the soft tissues, cartilage and bone of the hip joint [3]. Delayed diagnosis and treatment will eventually lead to adverse outcomes such as hip osteoarthritis and femoral head avascular necrosis. Children's hip joints still have some potential for development [4]. Open reduction, pelvic osteotomy combined with proximal femoral derotation, and varus/shortening osteotomy are often used as standard treatment for children with DDH who are ineffective in conservative treatment or late diagnosis [5].

The hip joint has a complicated anatomy, and the patient's hip joint has varying degrees of deformity. The only way to ensure a precise osteotomy during surgery to achieve good coverage after acetabular rotation and accurate correction of femoral derotation, varus and shortening osteotomies is to fully understand the degree of deformity before surgery and to formulate a precise surgical plan. Conventional surgery is planned preoperatively by assessing the severity of the deformity with preoperative pelvic and femoral radiographs or CT scans. During surgery, the osteotomy is performed under C-arm fluoroscopy using anatomical markers. The surgical outcome depends on the experience of the surgeon. Gallien et al. conducted a clinical and radiological study of the surgical outcome of 39 patients (43 hips) treated for congenital hip dislocation using open reduction combined with osteotomy and found that even experienced operator did not ensure the desired surgical outcome (18% failure rate) [6]. There is therefore an urgent need for more accurate and tailored treatment for children with DDH.

The use of 3D-printed technology in orthopedic clinics is becoming increasingly popular as a result of rapid advances in computer science and imaging technology. This has had a significant impact on how orthopedic diagnoses and treatments are now carried out. Research has shown that the use of 3D-printed surgical navigation template can help orthopedic patients to receive tailored and precise surgical care [7, 8]. Before surgery, three-dimensional software was used to create a precise surgical plan and intuitively understand the deformity. The design of the guide plates is carried out in the 3D editing software according to the proposed preoperative plan, and the 3D printer is used to obtain the entity of the guide plates to be used intraoperatively. The surgeon simply needs to locate the bone marker fixed navigation template according to the preoperative plan; using the template as a guide, the surgeon can then choose the direction, angle and depth of the osteotomy or screw placement. Using this simple procedure, surgery precision is greatly improved, and operation time is reduced. However, there are very few reports of this technique being used to surgically treat children with DDH. This study aims to compare the application benefits of proximal femoral osteotomy between 3D-printed guide-assisted and traditional surgical therapy in children with DDH, in conjunction with clinical practice, and to assess its clinical application value by collecting patient data.

## Patients and methods

### Clinical data

Retrospective data collection was done on the clinical information of children with DDH who underwent open reduction, pelvic Salter osteotomy and femoral osteotomy between September 2010 and September 2020. The study's inclusion and exclusion criteria led to the selection of a total of 36 kids (36 hips) as study participants. The individuals were split into a guide plate group (16 cases) and a conventional group (20 cases), depending on whether the 3D-printed navigation template was employed to facilitate femoral osteotomy. All operations were performed by the same experienced surgeon.

This study was authorized by the Fourth Affiliated Hospital of Harbin Medical University's Medical Ethics Review Committee (Approval ID: 2023-YXLLSC-02) and complied with the Declaration of Helsinki's guidelines. The children’s parents received information about the study and gave their informed consent.

The following inclusion and exclusion criteria were met by the chosen subjects:

Inclusion criteria: (1) children with unilateral developmental hip dislocation; (2) complete case data; (3) no history of hip infection; (4) no obvious hip and knee flexion deformity; (5) no prior relevant treatment; (6) the treatment plan was an open reduction of the affected hip, pelvic Salter osteotomy combined with femoral osteotomy; (7) informed of the study and surgical plan and follow-up time ≥ 48 months.

Exclusion criteria: (1) children with bilateral developmental hip dislocation; (2) prior treatment in any form; (3) combination with other malformations; (4) preoperative complications such as femoral head necrosis; (5) unilateral hip dislocation caused by neuromuscular diseases or other definite causes; (6) incomplete case data.

### Methods

Every patient had preoperative pelvic X-ray (supine, both lower limbs straight, toes internally rotated, both heels apart) and hip CT scan (supine position, knee and ankle joints together, iliac wing scan to the middle of the calf).

In the conventional group, the department developed the surgical strategy following a discussion based on the examination results. Following a three-dimensional reconstruction of the CT scan results, the guide plate group created the surgical plan and designed and printed each patient's unique femoral osteotomy guide plate. For all patients, derotation and varus were necessary for the proximal femur, and some individuals also had a shortening osteotomy.

### Creation of the surgical guide plates

The Mimics 21.0 software (Materialise, Leuven, Belgium) was used to import the DICOM-formatted CT data of the patients in the guide plate group, produce a new bone mask and segment the bone mask of the operation area using commands such as region expanding and altering the mask. After further smoothing and editing, the model was imported into 3-Matic 11.0 (Materialise, Leuven, Belgium), where the femoral anteversion angle, the femoral neck-shaft angle and the height of the femoral dislocation were all measured in three dimensions.

**Fig. 1:**
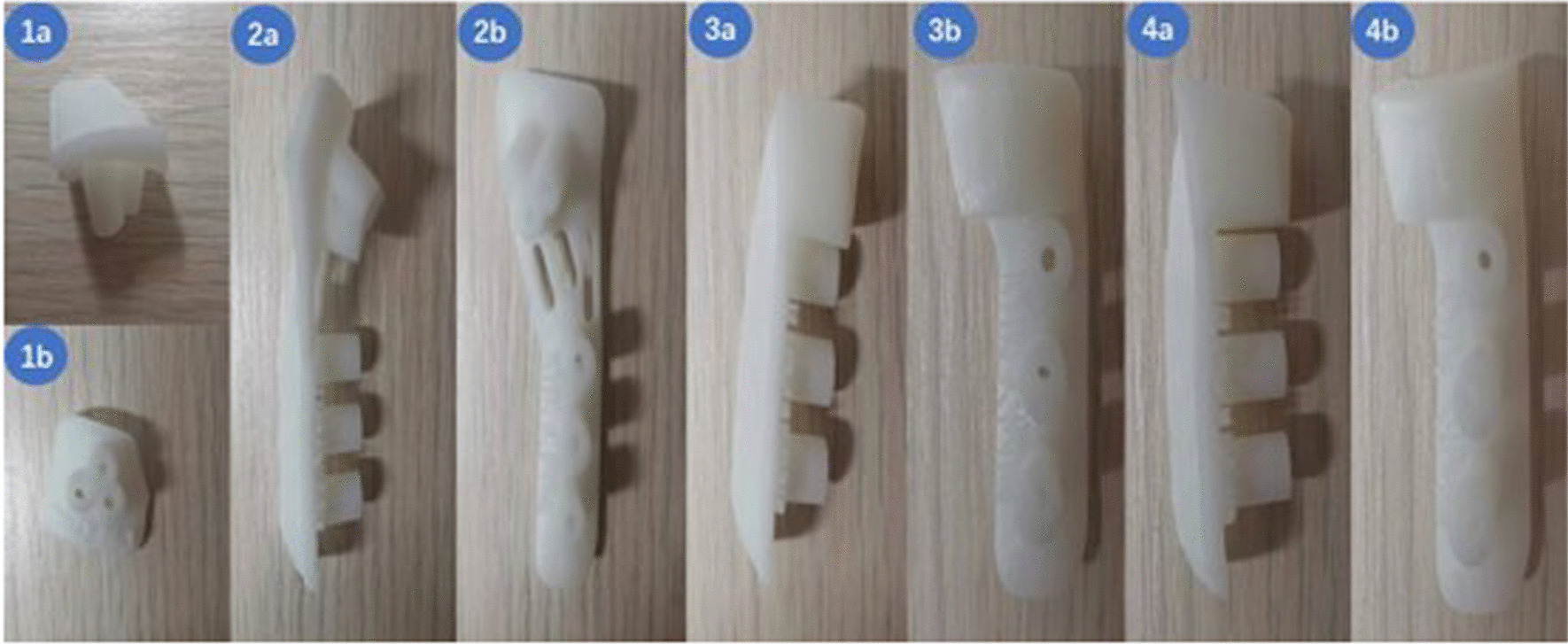
Lateral view (**a**) and front view (**b**) of the osteotomy guide plate object. (1) Positioning guide plate. (2) Connecting guide plate. (3) Transverse osteotomy guide plate. (4) Oblique osteotomy guide plate

The degree of varus, derotation/shortening osteotomy of the proximal femur, and measurement data were calculated, and the simulated operation was performed by the preoperative plan. The lower edge plane of the lesser trochanter was used as the horizontal osteotomy plane, and the greater trochanter served as the anatomical marker. Reverse engineering was used to create the lateral bone surface of the proximal femur, which was then exported in STL format using commands like consistency offset and Boolean operation. Third-party printing was commissioned after the design of the guides was completed (Fig. 1). (Note: The design of the guides was done by the treatment team and the cost of the third-party printing of the guides came from the subject funding. The patients did not have to pay for any costs related to the guides.)

### Operative procedure

#### Anesthesia, surgical exposure and Salter pelvic osteotomy

Children were positioned supine, with the affected side buttocks lifted to a pad height of 20° to 30°, and general anesthesia was given before endotracheal intubation. The Smith–Peterson method for the anterolateral hip was applied. To clean the acetabulum, the iliopsoas tendon was routinely severed, and the joint capsule was sliced in a "T" shape. The ischial notch, internal and external iliac plates, and other structures were visible. Using a pendulum saw, the osteotomy was carried out from the ischial notch above the anterior inferior iliac spine. After the osteotomy, two paper towel forceps were used to secure the distal and proximal ends of the osteotomy. To position the acetabular direction properly, the distal end of the osteotomy was rotated forward, downward and outward. From the iliac wing, a wedge-shaped bone block of the appropriate size was cut, positioned in the distal osteotomy's posterior area and securely fastened with Kirschner wire.

#### Proximal femoral varus, derotation/shortening osteotomy

On the outside of the proximal thigh, another longitudinal incision was created. Subperiosteal dissection exposed the greater trochanter of the femur and the lateral aspect of the upper femur.

Guide plate group: Four auxiliary osteotomy guides, including a positioning guide plate, a connecting guide plate, an oblique osteotomy guide plate and a transverse osteotomy guide plate, are needed for femoral osteotomies. The procedure was summed up as follows: Using a proximal femoral positioning guide, three Kirschner wires were placed; a connecting guide plate was installed; three distal Kirschner wires were placed; an oblique osteotomy guide plate was placed through three distal Kirschner wires; and a transverse osteotomy guide plate was placed through three distal Kirschner wires. It is possible to remove a wedge-shaped or trapezoidal bone block, depending on whether femoral shortening is carried out. The proximal and distal ends of the osteotomy are "face-to-face" involution after the bone block is removed [9].

Conventional group: In the pelvic X-ray and the hip CT, the bilateral neck-shaft angle and the anteversion angle, respectively, were measured. The varus and anteversion angle of the diseased side were planned to use the healthy side as the correction standard. The amount of distal movement needed to reduce the gap following horizontal osteotomy depends on the height of the femoral head dislocation in the distal femur.

**Fig. 2:**
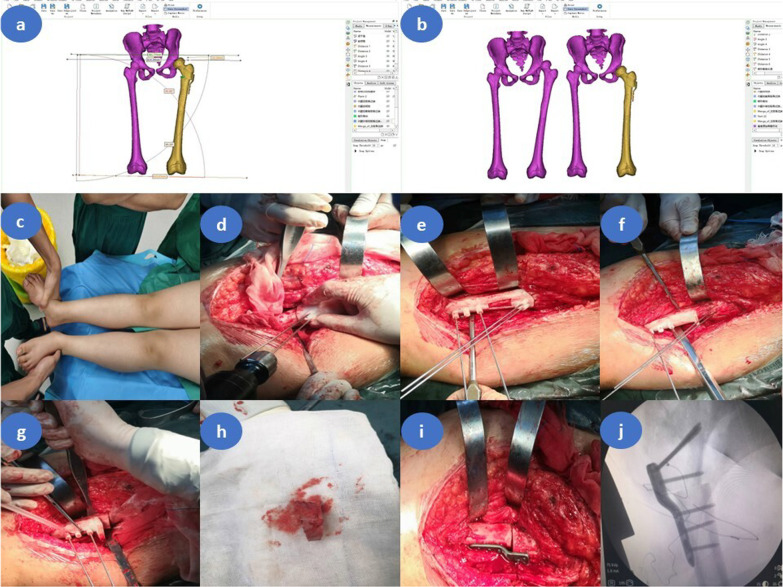
Intraoperative application of the osteotomy navigation templates in children with left developmental dysplasia of the hip (female patient, 5.5 years old, Tönnis type III). **a** Preoperative surgical planning in 3-Matic software. **b** Comparison of preoperative and postoperative CT scan three-dimensional reconstruction in 3-Matic software. **c** Both lower limbs are unequal, with the left shorter than the right. **d** Kirschner wires placement assisted by the positioning guide. **e** The distal Kirschner wires were properly inserted with the assistance of the connecting guide plate. **f** Implantation of the oblique osteotomy guide plate under the guidance of the distal Kirschner wires. The angle tilted cephalad to the osteotomy guide is the angle of varus of the proximal femur, and the first osteotomy was conducted by the swing saw with the aid of the guide. **g** The transverse osteotomy guide plate was placed under the guidance of distal Kirschner wires. The osteotomy surface of the guide plate was perpendicular to the femur, and the second osteotomy was conducted by the swing saw with the aid of the guide plate. The angle between the two osteotomy surfaces is the proximal femoral varus angle, and the length of the lateral femoral cortex between the two osteotomy surfaces is the additional shortening distance of the femur. **h** Trapezoidal bone block cutoff with the assistance of the guide plates. **i** LCP-PHP fixation of the distal and proximal femur. **j** C-arm fluoroscopy showed the plate was well-positioned and the anticipated orthopedic result was achieved

The locking compression pediatric hip plate (LCP-PHP) was used to fix the osteotomy's distal and proximal ends, and its proximal end was fixed to the greater trochanter epiphysis's lower edge (Fig. 2).

X-rays were used to assess the postoperative femoral neck-shaft angle and anteversion angle. (The lower limbs were fixed at 90° of knee flexion and hip flexion, the hip joint was abducted at 60°, the hips were symmetrical, the median axis of the femoral shaft was perpendicular to the median sacral crest on both sides and the angle between the Y line and the longitudinal axis of the femoral neck was the anteversion angle [10].)

#### Observational indicators

As intraoperative assessment indicators, operation time (total), operation time (femoral side), X-ray fluoroscopy times (total), X-ray fluoroscopy times (femoral side) and intraoperative blood loss were all employed. As postoperative evaluation indicators, postoperative neck-shaft angle, postoperative anteversion angle, hospitalization time and hospitalization expenses were all used. The clinical examination was carried out under McKay clinical criteria at the most recent follow-up. The evaluation content of McKay clinical criteria includes the stable state, pain, limp, range of motion and Trendelenburg sign of hip joint, which is divided into four grades: excellent, good, medium and poor [11]. All measurements are taken by the same observer to minimize measurement error.

### Statistical analysis

For the statistical evaluation of clinical data, the SPSS 26.0 software (IBM Corp, Armonk, NY, USA) was employed. The measurement data were presented as the mean ± SD. The outcomes of the data normality test were used in the intergroup comparison of measurement data. An independent-sample t-test was applied if the data followed a normal or roughly normal distribution; otherwise, a nonparametric rank sum test was applied. Chi-square test, corrected Chi-square test, or Fisher's precision probability test are all used to express count data as a rate. The difference was shown to be statistically significant at *P* < 0.05.

## Results

All 36 patients underwent open reduction, Salter pelvic osteotomy combined with proximal femoral derotation, and varus/shortening osteotomy satisfactorily. The surgical incisions healed in the first stage. Both the femoral osteotomy and pelvic bone graft sites recovered. During the follow-up period, no problems such as femoral head avascular necrosis, joint stiffness, or redislocation were discovered.

Preoperative neck-shaft angle (*P* = 0.429), preoperative anteversion (*P* = 0.266), follow-up time (*P* = 0.152), gender (*P* = 0.613), side (*P* = 0.731), Tönnis classification (*P* = 0.650) and age (*P* = 0.538) were not significantly different between the two groups (Table 1).

**Table 1:** Comparison of general demographic data and preoperative malformation between the guide plate group and the conventional group

General situation	Guide plate group (*n* = 16)	Conventional group (*n* = 20)	Statistics	*P* value
Age, years	4.38 ± 0.91	4.58 ± 0.98	*t* = − 0.623	0.538
Gender, *n* (%)			*	0.613
Male Female	1 (6.25) 15 (93.75)	3 (15.00) 17 (85.00)		
Side, *n* (%)			*	0.731
Left Right	11 (68.75) 5 (31.25)	12 (60.00) 8 (40.00)		
Tönnis classification, *n* (%)			*	0.650
II III IV	4 (25.00) 10 (62.50) 2 (12.50)	4 (20.00) 15 (75.00) 1 (5.00)		
Preoperative neck-shaft angle, °	146.49 ± 4.85	147.68 ± 4.04	*t* = − 0.801	0.429
Preoperative anteversion angle, °	45.26 ± 4.25	46.97 ± 4.66	*t* = − 1.132	0.266
Follow-up time, years	2.51 ± 0.38	2.70 ± 0.40	*t* = − 1.464	0.152

*Fisher's precision probability test

The results of the comparison of intraoperative observation indexes between the two groups were as follows: the operation time (total) of the guide plate group was shorter than that of the conventional group, 2.12 ± 0.13 h in the guide plate group and 2.69 ± 0.10 h in the conventional group, with a statistically significant difference between the two groups (*t* =  − 14.605, *P* < 0.001). The operation time (femoral side) was shorter in the guide plate group than in the conventional group, 0.39 ± 0.08 h in the guide plate group and 0.70 ± 0.08 h in the conventional group, with a statistically significant difference between the two groups (*t* =  − 11.635, *P* < 0.001). The X-ray fluoroscopy times (total) in the guide plate group were less than those in the conventional group, 4.50 ± 1.03 times in the guide plate group and 8.70 ± 1.03 times in the conventional group, and the difference between the two groups was statistically significant (*t* =  − 12.136, *P* < 0.001). The X-ray fluoroscopy times (femoral side) in the guide plate group were less than those in the conventional group, 2.56 ± 0.96 times in the guide plate group and 6.40 ± 0.99 times in the conventional group. The difference between the two groups was statistically significant (*t* =  − 11.660, *P* < 0.001). The intraoperative blood loss of the guide plate group was smaller than that of the conventional group. The guide plate group was 301.75 ± 31.61 ml, and the conventional group was 370.95 ± 35.82 ml. The difference between the two groups was statistically significant (*t* =  − 6.063, *P* < 0.001).

There was no significant difference between the guide plate group and the conventional group in postoperative neck-shaft angle (guide plate group: 121.31 ± 2.27°, conventional group: 122.41 ± 1.94°, *t* =  − 1.558, *P* = 0.129), postoperative anteversion angle (guide plate group: 15.68 ± 0.97°, conventional group: 16.03 ± 0.73°, *t* =  − 1.233, *P* = 0.226), hospitalization time (guide plate group: 12.25 ± 1.69 days, conventional group: 12.00 ± 2.00 days, *t* = 0.398, *P* = 0.693), hospitalization expenses (guide plate group: 28,817.06 ± 1645.26 yuan, conventional group: 29,586.75 ± 2038.49 yuan, *t* =  − 1.224, *P* = 0.229). At the last follow-up, the results of the Mackay clinical evaluation showed that 9 patients in the guide plate group were excellent, 5 patients were good, and 2 patients were fair; 9 patients in the conventional group were excellent, 7 patients were good, and 4 patients were fair. There was no significant difference (*P* = 0.742) between the two groups (Table 2).

**Table 2:** Comparison of surgical, postoperative and clinical evaluation data between the guide plate group and the conventional group

Observation indicators	Guide plate group (*n* = 16)	Conventional group (*n* = 20)	Statistics	*P* value
Operation time (total), hours	2.12 ± 0.13	2.69 ± 0.10	*t* = − 14.605	< 0.001
Operation time (femoral side), hours	0.39 ± 0.08	0.70 ± 0.08	*t* = − 11.635	< 0.001
X-ray fluoroscopy times (total), times	4.50 ± 1.03	8.70 ± 1.03	*t* = − 12.136	< 0.001
X-ray fluoroscopy times (femoral side), times	2.56 ± 0.96	6.40 ± 0.99	*t* = − 11.660	< 0.001
Intraoperative blood loss, ml	301.75 ± 31.61	370.95 ± 35.82	*t* = − 6.063	< 0.001
Postoperative neck-shaft angle, °	121.31 ± 2.27	122.41 ± 1.94	*t* = − 1.558	0.129
Postoperative anteversion angle, °	15.68 ± 0.97	16.03 ± 0.73	*t* = − 1.233	0.226
Hospitalization time, days	12.25 ± 1.69	12.00 ± 2.00	*t* = 0.398	0.693
Hospitalization expenses, yuan	28,817.06 ± 1645.26	29,586.75 ± 2038.49	*t* = − 1.224	0.229
Mackay clinical evaluation, *n* (%)			*	0.742
Excellent Good	9 (56.25) 5 (31.25)	9 (45.00) 7 (35.00)		
Fair	2 (12.50)	4 (20.00)		

^*^Fisher's precision probability test

## Discussion

DDH is a common hip deformity. Open reduction and pelvic osteotomy are performed to treat this disease, but often lead to complications such as avascular necrosis of the femoral head, redislocation and joint contracture [12, 13]. Clinical studies of combined proximal femoral osteotomy have been conducted by several scholars and have shown that femoral osteotomy significantly reduces the incidence of these complications [14–16].

However, proximal femoral deformities are usually complicated. Inadequate preoperative recognition of the deformity or inappropriate osteotomy can lead to complications such as non-union of the osteotomized segment, changes in the eccentric distance of the femur and postoperative redislocation [17]. Traditional surgical procedures are challenging, and the technical requirements are high, for the osteotomy of complicated bone abnormalities or complex bone anatomical features. According to experience, the surgeon can typically only make basic preoperative measurements and execute intraoperative osteotomies. Guaranteeing the osteotomy effect is not only arduous and time-consuming but also challenging [18, 19]. In a follow-up study of 55 children with DDH who presented with postoperative redislocation, Tang Chenglin et al. found that 13 cases were caused by the failure to correct the femoral deformity as expected [20]. A femoral varus osteotomy will reduce the femur's length. Both lower limbs may not be the same length in older patients because they have weaker adjustment abilities than younger patients. According to a study by Umer et al. [21], patients younger than 5 to 6 years old had better clinical results than older patients. This is also the most common complication of femoral shortening osteotomy. The long-term existence of unequal lower limbs will cause abnormal gait in patients, which will further lead to adverse consequences such as pelvic tilt, scoliosis and low back pain [22].

The outcome of an osteotomy is influenced by the patient's quality of life and even their prognosis. Because of the complexity of the lower limb structure, various planes and dimensions are frequently affected by the malformation. Surgery relies on the surgeon's subjective judgment, which can easily result in intraoperative osteotomy angle, placement and direction decision-making errors that compromise the precision and safety of the procedure. The maximum benefit can only be ensured by thoroughly understanding the deformity's features, conducting thorough preoperative planning, and carrying out the operation following the anticipated plan. Clinical orthopedics faces a significant issue in figuring out how to implement osteotomy with precision, individualization and standardization.

Computer-assisted design has become a significant component of digital medicine because of the advancement of computer technology. We can review CT data on a personal computer and reconstruct it in various windows and perspectives with the aid of computer-assisted design technologies. Realize the measurement of length and angle in three dimensions; plan and simulate surgery before the procedure; build an internal plant and an osteotomy guide plate, among other things. The advancement of this technology in orthopedics allows the surgeon to create individualized and accurate osteotomy plans and perform three-dimensional reconstruction, accurate measurement, and simulated surgery on the patient's skeletal lesion site [23]. With the aid of 3D printed technology, the CT scan data enable the actual model to be made and the physical model created is extremely accurate [24, 25]. 3D printing offers a novel technique and concept for individualized, accurate and standardized orthopedic therapy [26–28].

The design and 3D printing of osteotomy guide plates are beneficial for carrying out the established procedure accurately, conveniently, and with the least amount of intrusion possible. The traditional orthopedic osteotomy procedure is challenging. It is frequently only finished by senior doctors, even in big tertiary hospitals. The learning curve for this kind of surgery is too high for junior doctors with limited training. In comparison with conventional surgery, the use of osteotomy guide plates improves surgical outcomes while also lowering the operating threshold, making orthopedic osteotomy easier for junior surgeons to learn and use. The steps of the procedure can be made simpler, and the procedure time reduced by using the 3D-printed osteotomy guide plates. Honigmann et al. performed guide plates and conventional osteotomy in patients with wedge osteotomy of distal radius. The average operation time was 60 min in the 3D printing group compared to 90 min in the conventional group [29]. Our study found that 3D-printed guides significantly reduced operative time, decreased bleeding and lowered radiation exposure, but there was no statistically significant difference between the two groups in terms of postoperative measurements and evaluation at follow-up. This result may be due to the small sample size of the study, and we will next observe this result in a large-scale randomized controlled trial.

3D printing technology has a lower barrier to application compared to other new orthopedic technologies. The CT image data required for the design of the guide plates are available at all levels of hospital. Even in remote areas and primary care institutions, the design and printing of surgical guides can be commissioned from third-party companies to assist in traditionally difficult orthopedic operations, which not only benefits patients, but also helps to equalize medical resources and conditions. A novel approach to performing osteotomies is offered by computer-aided design, and 3D-printed osteotomy guide plates have a lot of application prospects.

Although the advantages of the application of the guide plates have been recognized by clinical practice, the number of large hospitals where this technology is widely available is also in the minority. The reasons for this are the lack of awareness of the technology among doctors, the lack of policies and the increase in medical costs. Brief clinical surveys have shown that even though 3D printing technology has been proven, most surgeons do not have a proper understanding of it. Some senior surgeons believe that they are too skilled to use other tools to demonstrate their profound surgical skills; some young surgeons consider this technology so advanced that it cannot be applied casually; and only a few surgeons who truly understand 3D printing have made this technology their right-hand man and promoted the development of this technology. Therefore, there is a great need to strengthen the popularization of 3D printing technology and unravel its mystery with the help of successful clinical cases, so that it can actually provide convenience for clinical practice. China identified 3D printing technology as an emerging strategic industry in 2013 and introduced several supporting policies to provide policy assurance for the application of 3D printing technology in clinical practice. Unfortunately, the corresponding medical service charges and medical insurance payment standards are extremely irregular. Only some provinces in the country have announced the fees for 3D printing medical services, but the specific names of the fees set by these provinces and the scope of implementation vary greatly and are not complete; many provinces lack corresponding fees. We believe that with the support of national policies and through the preliminary exploration of some provinces, the formulation of relevant policies and fee standards will become more and more perfect and unified. In our study, the design and printing of the guides took approximately 10 h. Although our study did not provide a clear figure for the cost, we roughly arrived at a price of approximately RMB 2,500 (approximately US$360) for the guides needed in our study, based on our calculations of labor, time and printing. The difference of hospitalization expenses between the two groups in our study was not statistically significant, since the guide plates were not designed and used at the corresponding cost to the patients. However, there was a significant advantage in the guide plate group in terms of shorter operative time and reduced bleeding, patients had less to spend on anesthesia, blood transfusions, C-arm photography, etc. Patients in the guide plate group had slightly lower costs than those in the conventional group, and by reducing the number of C-arms taken the patients had less exposure to radiation of medical origin, which would also benefit the patients. As national policies improve and 3D printing technology and equipment become more widespread, we believe that the cost of the guides will certainly drop significantly soon. In the meantime, we are working on developing an intelligent design and printing software for the guides, which will facilitate the promotion of this technology and reduce the cost.

**Fig. 3:**
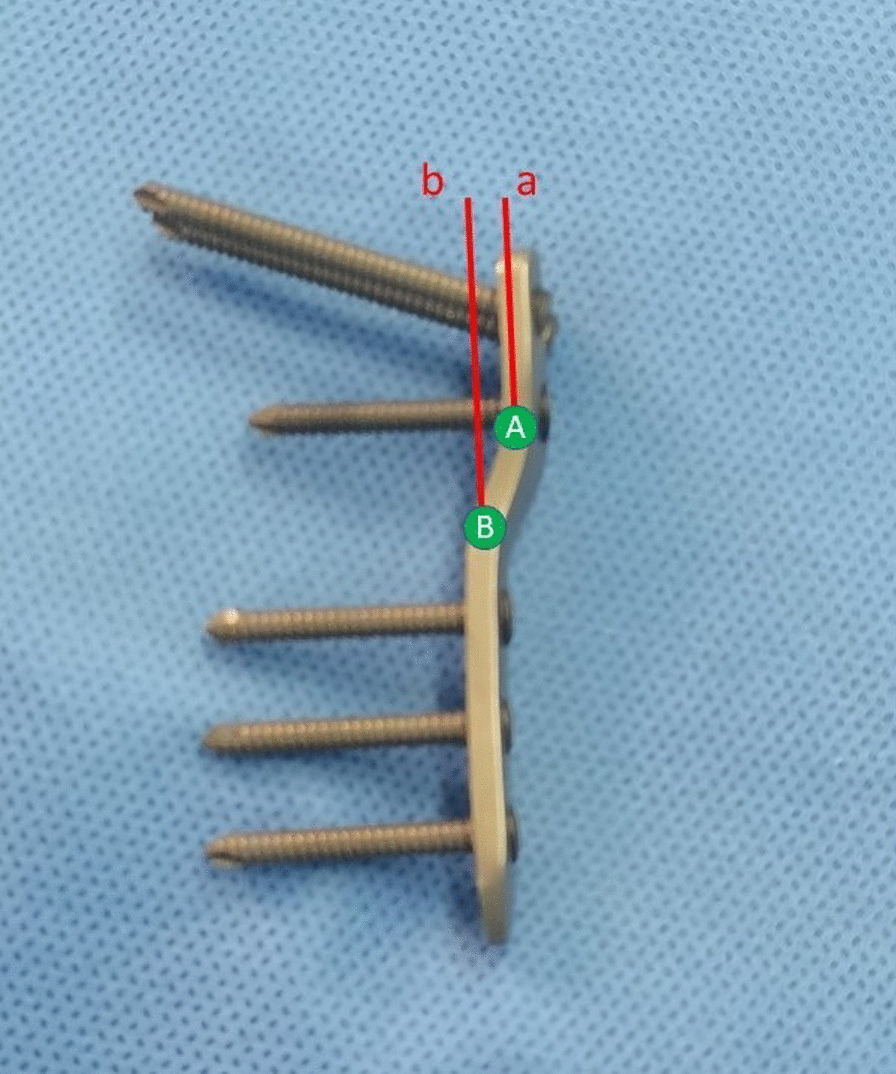
The distance between parallel lines a and b is the outward movement distance of the proximal femur after varus reserved by LCP-PHP (120°). Point A is the intersection of the proximal plate and the connecting part. Point B is the intersection of the distal plate and the connecting part. Parallel lines a and b are straight lines parallel to the femoral shaft through point A and point B respectively

**Fig. 4:**
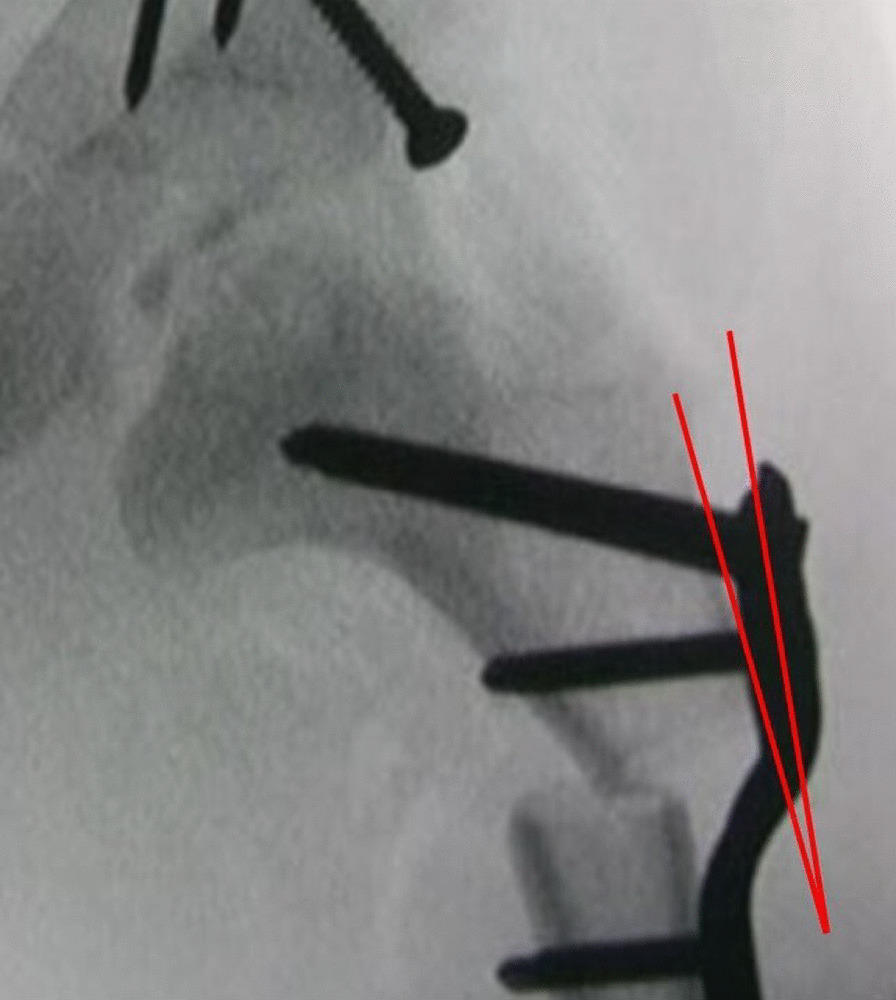
After fixation of the PHP-LCP, without displacement of the proximal femur, an angle is formed between the proximal plate and the proximal femoral cortex and where they do not adhere well together

Orthopedic surgeons attach great significance to the restoration of the lower limb alignment in the management of knee deformities and total knee arthroplasty, as it is essential for improving patients' symptoms and achieving surgical outcome. However, few reports have been published on preoperative and postoperative lower limb alignment changes in children with DDH, particularly in children in subluxation and complete dislocation, where the lower limb alignment is significantly shifted. The lower limb alignment also changes with the position of the femoral head after proximal femoral varus, which theoretically shifts the alignment inward. The LCP-PHP is used to fix the distal and proximal femur after osteotomy. It allows for a certain distance of outward movement of the proximal femur for force line recovery, as shown in Fig. 3, with two parallel lines a and b parallel to the femoral shaft at points A and B. The distance between the parallel lines a and b is the maximum distance of outward movement of the proximal femur. However, the distance that the proximal femur needs to be moved is different for children of different heights and different angles of proximal varus. The LCP-PHP is manufactured by the instrumentation company and cannot be used for personalized fixation. Theoretically, the distance of outward movement of the plate may be too large or too small for some patients. At the same time, the proximal plate is parallel to the femoral shaft. When the proximal femur is inwardly turned the proximal femoral cortex forms an angle with the proximal LCP-PHP and does not adhere well to the plate (Fig. 4). Tightening the plate proximal locking screw can easily pull the proximal femoral cortex toward the plate, which results in the loss of the required angle of varus for correction. Therefore, the same size of LCP-PHP currently used still has some shortcomings for clinical application. We will be investigating this issue with the aim of designing a customized and individualized plate and further validating its clinical value.

However, there are several shortcomings and problems in our study that still need to be resolved. (1) The follow-up period is brief; as avascular necrosis of the femoral head following surgery may not emerge for several years, the experimental results cannot accurately reflect the problems and long-term operation effects of the guide plate group and the conventional group. (2) Only the femoral side of the guide plate application is used in children who have improved postoperative hip function; the pelvic osteotomy effect is also important to note. The surgeon in this therapy group has extensive clinical expertise and has executed almost 100 pelvic osteotomies. The findings of this study also imply that there is no appreciable distinction between the conventional group and the guide plate group in terms of how femoral osteotomy affects them. Even yet, it is possible to underestimate the guide plate's application effect, and the use of the osteotomy guide plate during femoral and pelvic osteotomies may have distinct clinical outcomes. (3) Since the study was retrospective in nature, the reliability of the data is not as strong as it would be in a randomized controlled trial. For further confirmation, a large sample or multicenter randomized controlled trial is required.

## Conclusions

This study showed that 3D-printed osteotomy guide plates supporting proximal femoral osteotomy in children with DDH have the benefits of an uncomplicated operation, decreased operation time, reduced blood loss and intraoperative radiation exposure, and high clinical application value. The development of the 3D-printed guide plates and the advantages of its intraoperative use are comparable across all types and levels of difficulty, despite the reality that we only reviewed one orthopedic operation. Therefore, computer-aided design and 3D-printed technologies have broad therapeutic applications and can be used for other challenging surgeries.

## Data Availability

The datasets used and/or analyzed during the current study are available from the corresponding author on reasonable request.

## Abbreviation

DDH: Developmental dysplasia of the hip
